# Microbiome and photoperiod interactively determine thermal sensitivity of polar and temperate diatoms

**DOI:** 10.1098/rsbl.2023.0151

**Published:** 2023-11-15

**Authors:** Jakob K. Giesler, Tilmann Harder, Sylke Wohlrab

**Affiliations:** ^1^ Section Ecological Chemistry, Alfred Wegener Institute, Helmholtz Centre for Polar and Marine Research, 27570 Bremerhaven, Germany; ^2^ Marine Chemistry, Department of Chemistry and Biology, University of Bremen, 28359 Bremen, Germany; ^3^ Helmholtz Institute for Functional Marine Biodiversity at the University of Oldenburg (HIFMB), 23129 Oldenburg, Germany

**Keywords:** phytoplankton, microbiome, holobiont, diatom, light, temperature

## Abstract

The effect of temperature on ectothermic organisms in the context of climate change has long been considered in isolation (i.e. as a single driver). This is challenged by observations demonstrating that temperature-dependent growth is correlated to further factors. However, little is known how the chronobiological history of an organism reflected in its adaptation to re-occurring cyclic patterns in its environment (e.g. annual range of photoperiods in its habitat) and biotic interactions with its microbiome, contribute to shaping its realized niche. To address this, we conducted a full-factorial microcosm multi-stressor experiment with the marine diatoms *Thalassiosira gravida* (polar) and *Thalassiosira rotula* (temperate) across multiple levels of temperature (4°C; 9°C; 13.5°C) and photoperiod (4 h; 16 h; 24 h), both in the presence or absence of their microbiomes. While temperature-dependent growth of the temperate diatom was constrained by short and long photoperiods, the polar diatom coped with a 24 h photoperiod up to its thermal optimum (9°C). The algal microbiomes particularly supported host growth at the margins of their respective fundamental niches except for the combination of the warmest temperature tested at 24 h photoperiod. Overall, this study demonstrates that temperature tolerances may have evolved interactively and that the mutualistic effect of the microbiome can only be determined once the multifactorial abiotic niche is defined.

## 1 Introduction

Rising ocean temperatures lead to a global reorganization of species that move to track their thermal habitat. Accordingly, numerous studies of thermal adaptation capacities at different levels of biological organization have been published and used to estimate species vulnerability to global warming. While this research provided valuable insights into potential future range shifts and consequences at different ecological scales [1–3], the multifactorial nature of changing environmental conditions on temperature-driven range shifts has been largely neglected [4,5]. For example, diurnal and annual latitudinal light regimes are one of the most stable environmental signals, and chronobiological adaptations may limit the capacity for thermal range shifts due to photic mismatches, especially in photosynthetic organisms whose light regimes have imprinted a strong signal in the adaptive history and consequently coordinate various cellular functions [4,6].

Similarly, it is rarely considered in the context of temperature-driven range shifts that species do not occur as isolated entities and that their adaptations have evolved in concert with species interactions. On a small scale, a species interacts with its microbiome in complex and dynamic ways. Thus, a species' microbiome reflects a tight association in which the holobiont may have evolved to respond in a coordinated manner to changing conditions, often increasing the host fitness and resilience [7–9]. However, beneficial associations to cope with environmental conditions beyond the range of the evolved mutualism can be reversed, thereby amplifying negative effects, which can even cascade to higher levels of the respective biological system [10–14].

The goal of this study was to determine if diatom thermal performance is interactively affected by the organism's microbiome and the locally evolved chronobiology (i.e. tolerance to different photoperiods). To address this, we monitored growth of a temperate and a polar strain of the marine diatom *Thalassiosira* spp. in response to a gradual combination of temperatures and photoperiods both in the presence and absence of their native microbiomes.

## 2 Methods

### (a) Cultures and culture conditions

The polar *Thalassiosira gravida* (central Arctic Ocean) was obtained from the Norwegian culture collection of algae (NORCCA strain number UIO 478). The temperate *Thalassiosira rotula* from the German bight, was provided by the Harder Lab (University of Bremen; strain *T. rotula*_S16). Both strains were identified by their ITS1 sequences (electronic supplementary material, figure S1) and an axenic culture was rendered following the protocol of Mönnich *et al*. [15]. Axenicity of a culture was referred to as continuous absence of any contaminants stained with SYBR Green by regular (i.e. every 5–7 days) epifluorescence microscopy at 400× magnification (electronic supplementary material, figure S2). All cultures were maintained in climate chambers at a photoperiod of 16 : 8 h, a light intensity of 50 µmol photons m^−2^ s^−1^ and a temperature of 4°C and 15°C for the polar and temperate strains, respectively. Cultures were grown in filter-sterilized artificial seawater medium (ESAW) containing 1/5 of the vitamin concentration proposed by Harrison *et al*. [16] and kept in exponential growth by semi-continuous dilution.

### (b) Temperature–photoperiod growth assay

The growth of axenic and xenic strains of *T. gravida* and *T. rotula* was studied under multifactorial combinations of photoperiod [4 h; 16 h; and 24 h at 50 µmol photons m^−2^ s^−1^] and temperature [4°C; 9°C and 13.5°C] with the chosen levels based on previously assessed fully resolved thermal reaction norms. Cultures were pre-acclimatized to experimental conditions for a fixed acclimatization period of 7 days by inoculating 40 ml batch cultures with 500 cells ml^−1^ (2000 cells ml^−1^ for treatments with 4 h photoperiod to obtain sufficient biomass) which were grown at each of the nine treatment conditions to allow acclimatization of fluorophores. The actual subsequent multifactorial experiment was conducted in white 96-well plates (Greiner, Germany) with 300 µl experimental units and 48 replicates per treatment. After chlorophyll-a fluorescence of the acclimatized stock cultures was measured with a photo-spectrometric plate reader (ClarioStar Plus BMG Labtech, excitation 440 nm, emission 680 nm) plates were inoculated at twice the initial fluorescence units of the blank value measured in the growth medium. To maintain sterile conditions, the 96-well plates were sealed with a gas-permeable membrane (Breathe-Easy, Sigma-Aldrich, USA). Plates were incubated in climate cabinets at the respective experimental temperature and were placed onto LED tables emitting 50 µmol m^−2^ s^−1^ under the photoperiod settings above. Fluorescence intensity was measured daily at the same time after not more than 5 min of dark acclimation to not disturb photoperiod as an experimental factor. The experiment was terminated after 7 days.

### (c) Statistical analysis

After blank values of fluorescence in the growth medium were subtracted from the raw experimental fluorescence data, maximum growth rates (µ_max_) were calculated for each experimental unit by fitting nonlinear models to the data using the ‘growthrates’ package [17]. To test for main and interactive effects, a three-way ANOVA (type III SS) was conducted for *T. gravida* and *T. rotula*, respectively, with µ_max_ as dependent, and temperature, photoperiod and the presence/absence of the native microbiome as independent variables. Groups were weighted by the inverse of their variance to account for heteroscedasticity. *Post-hoc* analyses were conducted with Games–Howell tests. The effect sizes of the main effects and the three-way interaction were calculated with the ‘variancePartition’ package [18] which reports the fraction of explained variance to be attributed to each variable while correcting for all other variables. All statistical analyses and graphs were performed with the R environment 4.2.2 [19].

## 3. Results

For both, the polar and temperate diatom strain, temperature, photoperiod and the presence/absence of their respective microbiome had significant main effects on maximum growth rate, and showed a statistically significant three-way interaction (*p* < 0.001). The effect sizes of the independent variables differed largely in treatments with temperate and polar diatoms strains (table 1).

**Table 1.** ANOVA results and effect sizes of temperature, photoperiod and presence/absence of diatom microbiome on maximum growth rates (µ _max_) of *T. gravida* and *T. rotula*. Degrees of freedom (*d.f.*), *F* and *p*-values are given for each effect. Asterisk (*) indicate significant effects (*p* < 0.05). Effect sizes are given as % fraction of the total variance attributed to each factor.

effect	*T. gravida* (polar)				*T. rotula* (temperate)		
	*d.f.*	*µ*_max_			*µ*_max_		
		effect size	*F*	*p*	effect size	*F*	*p*
temp.	2	2.2	442.4	<0.001*	35.7	5444.2	<0.001*
photoperiod	2	45.7	5778.4	<0.001*	33.9	2837.3	<0.001*
bacteria	1	18.3	2381.5	<0.001*	11.9	2080.4	<0.001*
temp. × photoperiod × bacteria	4	31.2	377.2	<0.001*	16.6	448.2	<0.001*

For the polar strain, the temperature main effect explained 2.2% of the total variance in the dataset, but adding the factors of photoperiod and microbiome presence increased the explained variance by 45.7% and 18.3%, respectively. Thus, all main effects explained the variance by 66.1%. The analysis revealed an interactive effect of all terms, i.e. the effect of photoperiod and bacteria on temperature-dependent growth was not uniform. Including this interactive effect increased the explained variance by 31.2%, finally explaining 97.3% of the variability in the dataset.

For the temperate strain the temperature main effect accounted for 35.7% of the variance, while the effect size of photoperiod and microbiome presence explained 33.9% and 11.2% of the variance, respectively. Also, for the temperate diatom the maximum growth rate was not only affected by additive main effects, but also by their interactive effect, explaining 16.6% of the variance, adding up the total explained variance to 97.4%.

**Figure 1.**
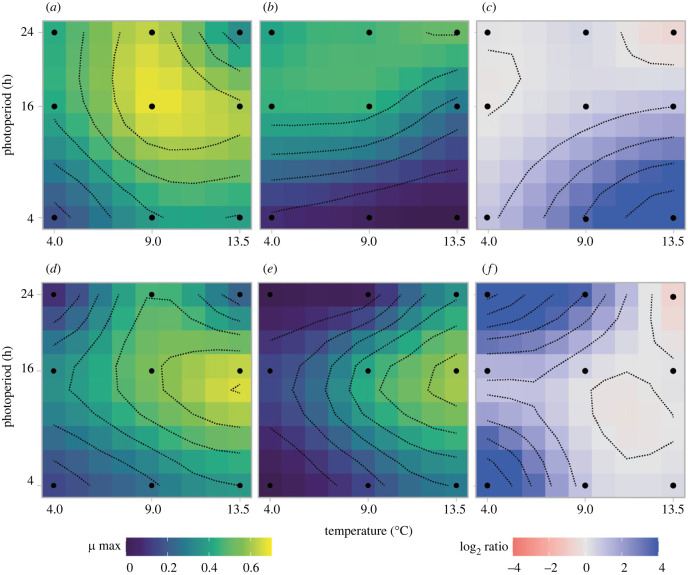
Maximum growth rates for the polar *T. gravida* (upper panel) and the temperate *T. rotula* (lower panel). Interpolated response surface plots of the effects of temperature and photoperiod on (*a,d*) maximum growth rates (µ_max_ day^−1^ indicated by colour) of the xenic strain and (*b,e*) the axenic strain as well as (*c,f*) the log_2_-response ratio (LRR indicated by colour) of xenic divided by axenic maximum growth rates displaying positive (blue) and negative (red) microbiome effects. Black lines indicate isolines of the displayed variable (bin width of 0.1 for µ_max_ and bin width of 1.2 for the log-ratio). Black dots represent tested experimental conditions.

In terms of the direction of these effects, the xenic polar diatom had its optimum growth conditions at 9°C and a 16 h photoperiod (figure 1*a*). The axenic polar diatom strain did not show this clear optimum and revealed decreased maximum growth rates with increasing temperature and decreasing photoperiod (figure 1*b*). This growth pattern was also reflected in the log response ratios where the difference in maximum growth rates between the xenic and axenic polar strain increased with increasing temperature and decreasing photoperiod (figure 1*c*). Although the xenic polar strain generally had higher maximum growth rates than the axenic strain, this pattern was reversed at 13.5°C in combination with a 24 h photoperiod. Here, the axenic polar strain showed significantly higher maximum growth rates than the xenic strain (*p* < 0.001).

For the temperate diatom, both the xenic and axenic strains exhibited highest growth rates at 13.5°C under a 16 h photoperiod (figure 1*d,e*). The xenic temperate diatom showed overall higher maximum growth rates than the axenic strain, especially at 9°C and a 24 h photoperiod (figure 1*f*). Yet, just like for the polar diatom, at 13.5°C and a photoperiod of 24 h, the axenic temperate strain showed significantly higher maximum growth rates than the xenic strain (*p* < 0.001).

## 4 Discussion

Both the polar and temperate *Thalassiosira* strains showed specific adaptations to their respective chronobiological and climatic geographical history, reflected in their growth response across multiple levels of temperature and photoperiod. This study revealed that the presence of the diatom microbiome enhanced maximum diatom growth rates especially at the margins of their respective niche, except for the warmest temperature under the 24 h photoperiod.

While the temperate axenic and xenic diatom did not reach their thermal optimum in this experimental design, the polar (xenic) diatom displayed its highest growth rates at 9°C, a water temperature rarely reached in the Arctic Ocean [20]. However, it was demonstrated by Thomas *et al*. [21] that especially at high latitudes, planktonic organisms do not live at their thermal optimum but rather at the onset of their temperature reaction norm (unimodal, left-skewed function describing an ectothermic organism's growth/fitness in response to temperature). This may be beneficial to survive heat waves, and is also logical as the fundamental niche is constrained by further drivers shaping the organism's realized niche. For example, high growth rates at elevated temperatures may be associated with unsustainable resource depletion, i.e. the two factors may not scale linearly. In a natural environment, this limits the allocation of energy to crucial pathways that maintain growth in response to multiple abiotic or biotic factors, and maximum growth may therefore not pay off [22,23].

Interactive effects of light and temperature on photosynthesis are comparatively well understood in plants. Here, prolonged photoperiods exacerbate the negative effects of elevated temperatures on photosynthesis by triggering oxidative burst-like reactions that additionally affect temperature-impaired electron transport capacities [24]. Our study showed that the polar strain coped with short and long photoperiods until its temperature optimum was reached. By contrast, the temperate strain was more sensitive to 24 h light exposure as its growth rate was significantly affected prior to the optimum temperature. Biogeographically determined adaptations of the strains, such as photoperiod-dependent physiology and cell division regulated by the molecular circadian clock, may cause the observed growth rate patterns. The more flexible response of the polar compared to the temperate diatom to the photoperiods tested may therefore be linked to their evolved chronobiology. Diverse polar species, e.g. marine zooplankton [4], fruit flies [25] and reindeer [26], were found to have plastic molecular clocks, whereas their temperate representatives remain entrained in their rhythmicity [25,26]. While this flexibility has not been reported for polar diatoms, entrainment in rhythmicity is at least known for the temperate diatom *Phaeodactylum.* Here, rhythmic gene expression of a key circadian clock regulator and associated cellular functions persist under constant light (and dark) conditions [27]. This supports the conclusion that due to the synergistic nature of light and temperature stress, in tandem with the control of light stress by the circadian rhythm, the chronobiological background of the strains tested did affect their sensitivity to temperature. With regard to the microbiome, this is further supported by studies that found diel cycle dependent patterns of highly coordinated gene expression [28] and metabolite production [29] between the host and its microbiome for key resources shared within the phytoplankton holobiont.

Bacteria provide various services to diatoms which impact their growth and survival, such as nutrient recycling [30], biofilm formation [31] or the synthesis of vitamins [32] and growth stimulating compounds [33]. The present study provides evidence that the positive net effects of the microbiome were not equally distributed across multiple temperature and photoperiod levels. Hence, the growth supporting effect of the microbiome come into play at specific environmental conditions that are at least biogeographically determined, if not even genotype-specific [34,35]. For the polar xenic diatom, the growth supporting effect occurred especially at temperatures above its thermal optimum in combination with short photoperiods. For the xenic temperate diatom, the microbiome enhanced its growth towards its thermal minimum, especially in combination with extreme photoperiods, i.e. the 4 h and 24 h photoperiod. Yet, it must be taken into account that the experimental units used in this study are a closed system that do not allow the assembly of a new microbiome, potentially more beneficial at specific environmental settings as demonstrated for plants and their soil microbiomes [36]. However, a prior study investigating the microbiome reassembly of 81 strains of *T. rotula* in a common garden experiment under consideration of environmental stressors, observed a much stronger association of the microbiome to its respective host's genetic population than explained with environmental factors alone [34]. This suggested an association of diatoms and their microbiomes for long time frames, up to decades, which thus may limit the capacity of host cells to recruit new microbiome bacteria.

Since DIC concentrations were not measured, the possibility of carbon limitation must be considered, especially in the axenic cultures in the absence of bacterial respiration. To reduce this possibility, the experimental duration was short and a very dilute inoculum was used. This is evidenced by the low starting fluorescence value, as recommended for nanocosm approaches according to Volpe *et al*. [37].

Since axenicity was verified by epifluorescence microscopy, the putative presence of bacteria on algal cells outside examined fields of view cannot be completely ruled out. However, the effect of these potential minimal contaminations on a diatom culture are considered to be negligible, firstly because bacteria-derived compounds affecting phytoplankton–bacteria interactions are concentration dependent [38,39], and secondly because bacterial densities in xenic cultures would be six orders of magnitude higher than minute bacterial contaminations in pseudo-axenic cultures [40].

In contrast to the observed growth enhancement in the presence of the microbiomes at the described conditions, both axenic diatom strains exhibited higher maximum growth rates than the xenic strains under a combination of 13.5°C and 24 h photoperiod. This indicates a reversal of the overall mutualistic relationship of the diatom microbiome at this environmental condition, which exceeds the pure loss of symbiotic biotic interactions, an observation known from different holobiont systems like plants [7], corals [41] or human holobionts [42], commonly referred to as microbiome dysbiosis [7]. Especially when exposed to several stressors, a stochastic reassembly of the hosts microbiome (Anna Karenina Principle) often encompassing bacterial taxa that reduce the hosts fitness, can occur [7]. For the polar diatom strain, the sensitivity to prolonged photoperiods increased at temperatures beyond its physiological optimum could therefore potentially increase the vulnerability to microbiome dysbiosis under these conditions. For the temperate diatom strain, this sensitivity to a prolonged photoperiod generally seemed to increase with higher temperatures already prior to the thermal optimum.

Considering the closed system design of this study, potential dysbiosis is limited either to a change in the relative composition of the microbiome (i.e. parasitic species are already present in the system at low abundance), or to a reversal of mutualistic relationships as demonstrated for opportunistic microbiome bacteria that first produce growth stimulating compounds for their host cell and eventually turn parasitic and switch to production of algicides when the fitness of their host is decreasing [43,44]. This underlines the importance to consider mutualistic relationships as context dependent.

The outcome of this study underlines that the thermal sensitivity of diatoms is an integrated response to multifactorial parameters. Specifically, temperature-dependent growth is interactively influenced by the photoperiod in a chronobiological context and biotic interactions, namely co-occurring microbiome bacteria. In addition, we demonstrated the importance of the microbiome in supporting growth of host diatoms, particularly under unfavourable conditions at the margins of their fundamental niches. We suggest that future studies of species adaptability should consider that tolerances are defined and emerge interactively. This is an important aspect for identifying critical thresholds, determining species resilience, and assessing potential adaptive capabilities. In particular, the question of which factor (temperature sensitivity, chronobiology, or biotic interactions) is more evolutionarily constrained is crucial for modelling future habitat shifts of arctic and temperate phytoplankton species. Detailed knowledge of their regulation and evolutionary history is therefore required to assess how future adaptive capacities are possible given the multifactorial nature of changing environmental conditions.

## Data Availability

All data and codes as well as their respective detailed descriptions (metadata) to fully reproduce this study are available from the Dryad Digital Repository: https://doi.org/10.5061/dryad.x95x69pqf [45]. Data available as part of the electronic supplementary material [46].
